# Suppression of Indoxyl Sulfate Accumulation Reduces Renal Fibrosis in Sulfotransferase 1a1-Deficient Mice

**DOI:** 10.3390/ijms241411329

**Published:** 2023-07-11

**Authors:** Huixian Hou, Mai Horikawa, Yuki Narita, Hirofumi Jono, Yutaka Kakizoe, Yuichiro Izumi, Takashige Kuwabara, Masashi Mukoyama, Hideyuki Saito

**Affiliations:** 1 Department of Clinical Pharmaceutical Sciences, Graduate School of Pharmaceutical Sciences, Kumamoto University, 1-1-1 Honjo, Chuo-ku, Kumamoto 860-8556, Japan; 202y2051@st.kumamoto-u.ac.jp (H.H.); maisiba0101@gmail.com (M.H.);; 2 Department of Pharmacy, Kumamoto University Hospital, 1-1-1 Honjo, Chuo-ku, Kumamoto 860-8556, Japan; 3 Department of Nephrology, Graduate School of Medical Sciences, Kumamoto University, 1-1-1 Honjo, Chuo-ku, Kumamoto 860-8556, Japan

**Keywords:** indoxyl sulfate, renal fibrosis, sulfotransferase 1a1-deficient mice

## Abstract

Renal fibrosis is the final manifestation of chronic kidney disease (CKD); its prevention is vital for controlling CKD progression. Indoxyl sulfate (IS), a typical sulfate-conjugated uremic solute, is produced in the liver via the enzyme sulfotransferase (SULT) 1A1 and accumulates significantly during CKD. We investigated the toxicopathological role of IS in renal fibrosis using *Sult1a1*-KO mice and the underlying mechanisms. The unilateral ureteral obstruction (UUO) model was created; kidney IS concentrations, inflammation, and renal fibrosis were assessed on day 14. After UUO treatment, inflammation and renal fibrosis were exacerbated in WT mice, with an accumulation of IS in the kidney. However, they were significantly suppressed in *Sult1a1*-KO mice. CD206^+^ expression was upregulated, and β-catenin expression was downregulated in *Sult1a1*-KO mice. To confirm the impact of erythropoietin (EPO) on renal fibrosis, we evaluated the time-dependent expression of EPO. In *Sult1a1*-KO mice, EPO mRNA expression was improved considerably; UUO-induced renal fibrosis was further attenuated by recombinant human erythropoietin (rhEPO). Thus, UUO-induced renal fibrosis was alleviated in *Sult1a1*-KO mice with a decreased accumulation of IS. Our findings confirmed the pathological role of IS in renal fibrosis and identified SULT1A1 as a new therapeutic target enzyme for preventing and attenuating renal fibrosis.

## 1. Introduction

Numerous epidemiological studies indicate that chronic kidney disease (CKD) prevalence is increasing globally [1,2]. Renal fibrosis is recognized as a final manifestation among patients with CKD, regardless of the initial causes; many patients eventually progress to end-stage kidney failure, a devastating condition that requires lifelong dialysis or kidney transplantation [3]. Despite the increasing number of patients with CKD, current clinical pharmacological therapies are scarce and ineffective. Although many studies have provided effective therapies for renal fibrosis established on animal models, an approved treatment for CKD patients is very few [4]. Thus, novel insights into the molecular mechanisms of renal fibrosis and therapeutic strategies are urgently needed.

Indoxyl sulfate (IS), a typical sulfate-conjugated uremic toxin [5,6], is thought to be a risk factor for renal disease progression because it accumulates in the body during renal dysfunction and causes oxidative stress [7]. IS in the blood circulation was absorbed into proximal tubular cells via the organic anion transporters OAT1 and OAT3 and then excreted into the urine [8]. When kidney function is compromised, IS accumulates significantly in the blood and kidney of the AKI model, leading to kidney damage [9]. Several clinical studies revealed that patients with CKD having high IS (≥6.124 mg/L) were significantly associated with renal progression to dialysis [10,11]. However, the effect of IS on renal fibrosis has not been widely investigated in a mouse model. In addition, IS removal by dialysis is difficult due to its high protein binding rate, particularly to serum albumin (∼95%) [12]. On the other hand, AST-120, a charcoal adsorbent, which adsorbs IS precursors in the gut, reduces serum IS levels in patients with CKD and inhibits CKD progression [13,14]. However, AST-120 requires large doses, making it challenging to maintain adherence; furthermore, it nonspecifically adsorbs concomitant medications [15], among other clinical problems. Consequently, it is acknowledged that developing new strategies to lower or remove the systemic accumulation of IS in patients with kidney disease and uremia is an urgent issue.

Here, we focus on the production of IS. IS is produced in the liver by CYP2A6/2E1-dependent oxidative metabolism of gut-derived indole, followed by sulfotransferase (SULT) 1A1-mediated sulfate transfer to indoxyl. We previously reported that the concentration of IS in the serum and kidney was lower in *Sult1a1*-deficient (*Sult1a1*-KO) cisplatin mice [16], and the increase of IS level could be due to the downregulation of renal organic ion transporters and central nervous system toxicities in cisplatin-induced AKI model rats [17,18]. Moreover, the inhibition of IS production elicited a renoprotective effect in the ischemic AKI model [9]. Notably, the in-depth mechanism of IS on progressive renal fibrosis has not been well demonstrated.

In this study, we investigated the toxicopathological role of IS in renal fibrosis using *Sult1a1*-KO mice and the underlying mechanisms.

## 2. Results

### 2.1. IS Accumulation Was Reduced in the UUO Model Using Sult1a1-KO Mice

**Figure 1:**
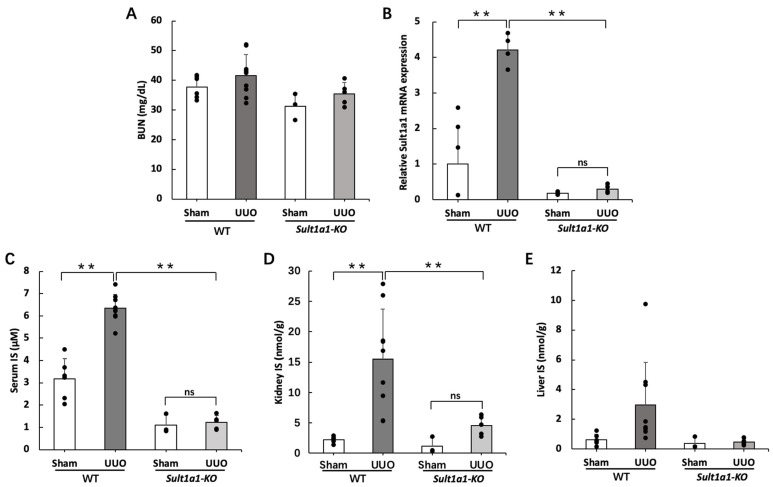
Effect of *Sult1a1*-KO on IS concentration in serum and kidney. (**A**) Serum BUN concentration in WT and *Sult1a1*-KO mice. (**B**) *Sult1a1* gene expression checked using RT-PCR. (**C**–**E**) IS concentration in serum, kidney, and liver calculated via LC-MS/MS. Each value represents the mean ± SD of 3–9 mice. ** *p* < 0.01, ns: not significant.

To confirm the effect of *Sult1a1* deficiency on IS accumulation, we first confirmed the renal function and the knockout condition of *Sult1a1*. We also assessed the concentration of IS in the serum, kidney, and liver. Serum BUN levels did not increase significantly in the WT UUO and *Sult1a1*-KO UUO groups (Figure 1A). *Sult1a1* mRNA expression increased in the WT UUO group and decreased significantly in the *Sult1a1*-KO UUO group (Figure 1B). Serum and renal IS concentrations were elevated in the WT UUO group but were relatively lower in the *Sult1a1*-KO UUO group (Figure 1C,D). An increasing trend in hepatic IS concentration was observed in the WT UUO group (Figure 1E). In contrast, a decreasing trend was observed in the *Sult1a1*-KO UUO group. These indicate that serum and kidney IS concentration was decreased in *Sult1a1*-KO mice even after UUO surgery, suggesting that hepatic Sult1a1 is involved in the production/accumulation of IS under UUO-induced kidney injury.

### 2.2. Gene Expression of Inflammation and Renal Fibrosis Improved in the Sult1a1-KO Mice UUO Model

**Figure 2:**
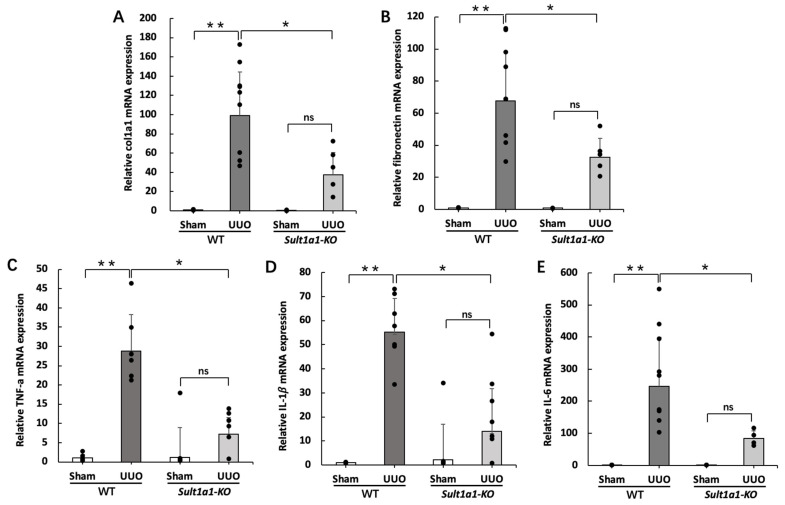
Effect of *Sult1a1*-KO on inflammation, renal fibrosis. (**A**,**B**) gene expression of renal fibrosis markers (col1a1, fibronectin) checked using RT-PCR. (**C**–**E**) gene expression of inflammatory cytokines (TNF-α, IL-1β, IL-6) checked using RT-PCR. Each value represents the mean ± SD of 3–9 mice. * *p* < 0.05, ** *p* < 0.01, ns: not significant.

To investigate the effect of *Sult1a1* deficiency in the UUO model, we examined the gene expression of renal fibrosis and inflammatory cytokines using RT-PCR. Renal fibrosis markers, col1a1 and fibronectin, were highly expressed in the WT UUO group but were significantly suppressed in the *Sult1a1*-KO UUO group (Figure 2A,B). Moreover, TNF-α and IL-1β gene expression increased significantly in WT UUO mice kidneys but were markedly suppressed in the *Sult1a1*-KO UUO group kidneys (Figure 2C,D). The expression of IL-6 demonstrated a similar tendency (Figure 2E), suggesting that UUO-induced inflammatory response and renal fibrosis were alleviated with decreased serum and renal IS accumulation.

### 2.3. Renal Fibrosis Is Suppressed in the UUO Model Using Sult1a1-KO Mice

**Figure 3:**
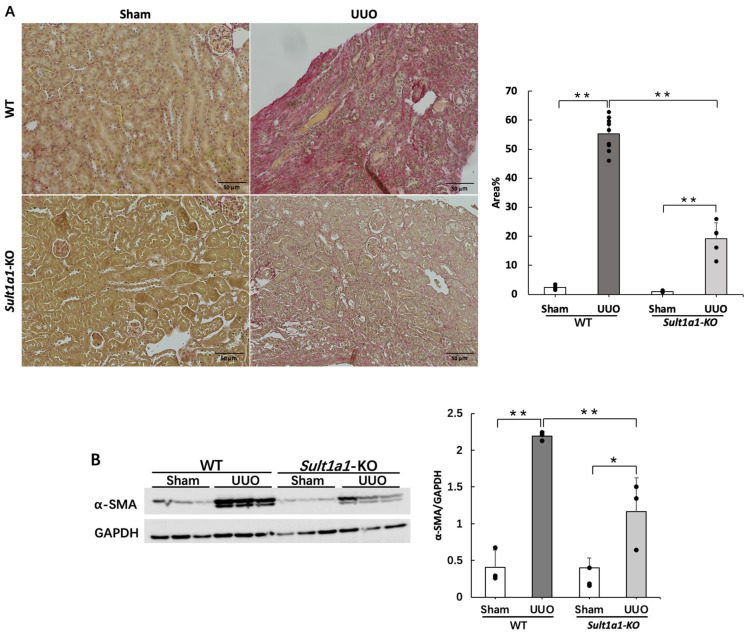
Effect of *Sult1a1*-KO on UUO-induced renal fibrosis. (**A**) collagen deposition (the red part) in the kidney confirmed by Sirius red staining. The scale bar = 50 μm. (**B**) Western blot experiment confirmed the expression of α-SMA. Each value represents the mean ± SD of 3–9 mice. * *p* < 0.05, ** *p* < 0.01.

We further confirmed the effect of *Sult1a1* deficiency on UUO-induced renal fibrosis using Sirius red staining and the protein expression of α-SMA. The UUO model exhibits prominent renal fibrosis. Thus, we examined whether UUO-induced renal fibrosis could be improved in *Sult1a1*-KO mice. Sirius red staining (Figure 3A) revealed marked collagen deposition (red part) in the kidneys of the WT UUO group, but were significantly suppressed in the *Sult1a1*-KO UUO group. Additionally, α-SMA protein expression, a myofibroblast marker (Figure 3B), demonstrated decreased fibrosis in the kidneys of *Sult1a1*-KO mice. These data indicated that UUO-induced renal fibrosis was significantly alleviated in *Sult1a1*-KO mice.

### 2.4. The UUO Model Using Sult1a1-KO Mice Allowed CD206^+^ Macrophage Infiltration and Reduced Apoptosis

**Figure 4:**
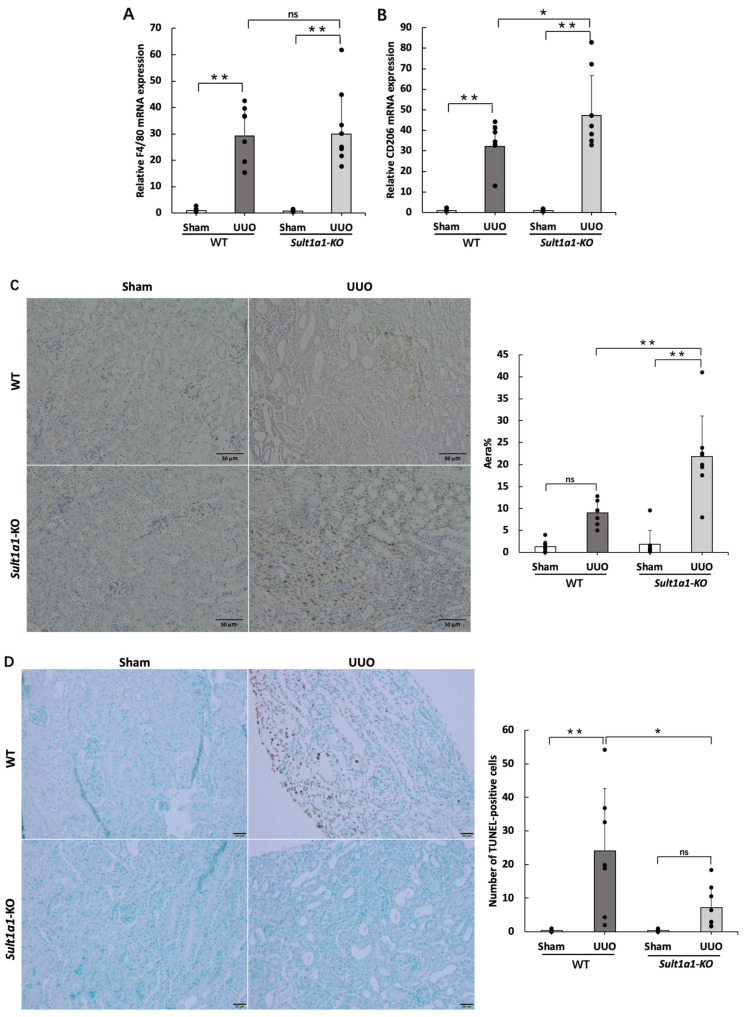
Effect of *Sult1a1*-KO on UUO-induced CD206^+^ macrophage infiltration. (**A**,**B**) gene expression of F4/80 and CD206^+^ checked using RT-PCR. (**C**) immunostaining of CD206. The scale bar = 50 μm. (**D**) apoptotic cells checked via TUNEL staining. The scale bar = 50 μm. Each value represents the mean ± SD of 7–8 mice. * *p* < 0.05, ** *p* < 0.01, ns: not significant.

We also evaluated macrophage activation because inflammatory cytokines showed significant differences between WT and *Sult1a1*-KO mice. F4/80 has been used widely as a marker for mouse macrophages. Although F4/80 demonstrated no significant differences between the WT UUO and *Sult1a1*-KO UUO groups (Figure 4A), CD206^+^, an anti-inflammatory macrophage-related surface marker, was significantly increased in the *Sult1a1*-KO UUO group (Figure 4B). Immunostaining for CD206^+^ macrophages revealed similar results, with significantly increased invasive areas in the *Sult1a1*-KO UUO group (Figure 4C). These results revealed that the infiltration of CD206^+^ macrophages could play a key role in attenuating inflammation and renal fibrosis in *Sult1a1*-KO UUO mice. Furthermore, apoptosis, confirmed with TUNEL staining, was significantly decreased in the *Sult1a1*-KO UUO group (Figure 4D), suggesting that apoptosis was suppressed with reduced IS accumulation.

### 2.5. The UUO Model Using Sult1a1-KO Mice Activated Wnt/β-Catenin Signaling

**Figure 5:**
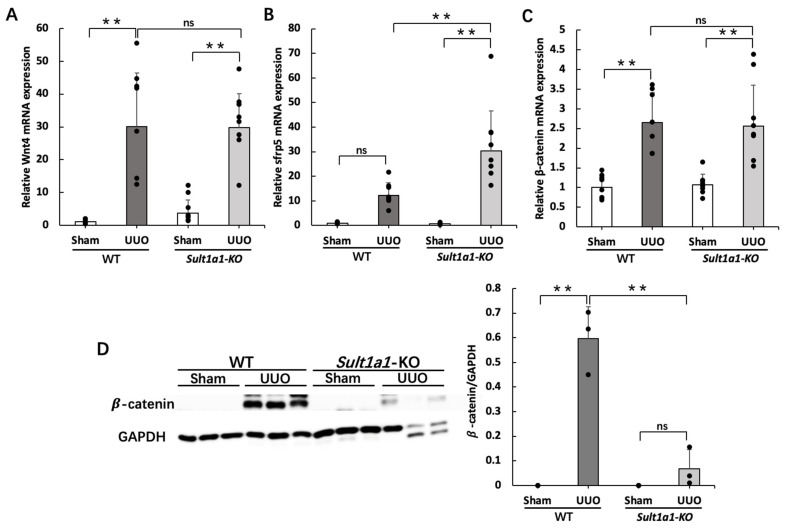
Effect of *Sult1a1*-KO on Wnt/β-catenin signaling activation. (**A**) gene expression of Wnt4 checked using RT-PCR. (**B**) gene expression of *Sfrp5* checked using RT-PCR. (**C**) gene expression of β-catenin checked using RT-PCR. (**D**) protein expression of β-catenin checked via western blot. Each value represents the mean ± SD of 7–8 mice. ** *p* < 0.01, ns: not significant.

Notably, Wnt/β-catenin signaling activation triggers tubular epithelial cell transition to mesenchymal or senescent phenotype and promotes renal fibrosis [19]. We considered whether the Wnt/β-catenin signal affects the attenuated renal fibrosis in *Sult1a1*-KO mice. Notably, while Wnt4 expression was comparable in the WT UUO and *Sult1a1*-KO UUO groups (Figure 5A), the expression of sfrp5, which acts as a Wnt protein antagonist, was significantly increased in the *Sult1a1*-KO UUO group (Figure 5B). Furthermore, despite similar gene expression of β-catenin in the UUO and *Sult1a1*-KO UUO groups (Figure 5C), protein expression was significantly decreased in the *Sult1a1*-KO UUO group (Figure 5D), suggesting that a portion of the Wnt/β-catenin signaling pathway was inactivated with decreased IS accumulation.

### 2.6. Administration of rhEPO Attenuated Renal Fibrosis in the UUO Model Using Sult1a1-KO Mice

**Figure 6:**
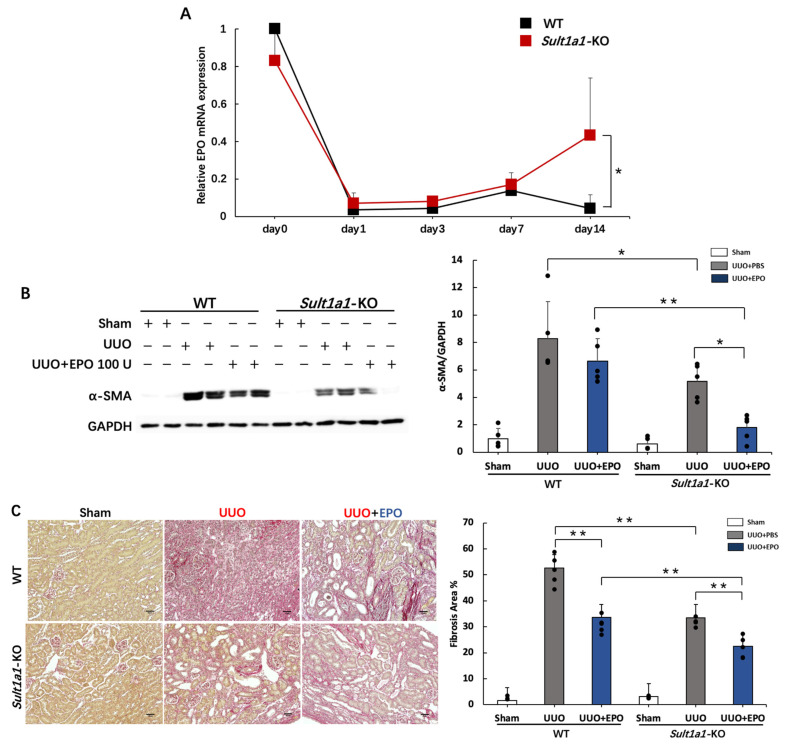
Effect of rhEPO treatment and *Sult1a1*-KO on renal fibrosis. (**A**) time-dependent expression of EPO gene expression tested using RT-PCR. (**B**) Protein expression of α-SMA checked using western blot. (**C**) Collagen deposition (the red part) in the kidney confirmed via Sirius red staining. The scale bar = 50 μm. Each value represents the mean ± SD of 3–8 mice. * *p* < 0.05, ** *p* < 0.01.

Renal EPO-producing cell plasticity governs fibrosis [20]. Therefore, we evaluated the time-dependent EPO mRNA expression. While EPO expression decreased in the WT UUO group after UUO surgery, it significantly improved by day 14 in the *Sult1a1*-KO UUO group (Figure 6A). To further investigate the role of EPO in IS-exacerbated renal fibrosis, we administered rhEPO to WT and *Sult1a1*-KO mice, as described in protocol 3. α-SMA increased in the WT UUO group but was significantly decreased in the *Sult1a1*-KO UUO group, and further decreased in the *Sult1a1*-KO UUO+EPO group treated with rhEPO (Figure 6B). Sirius red staining revealed similar results as those observed for α-SMA expression (Figure 6C), indicating that combining *Sult1a1*-KO and rhEPO treatment might prevent renal fibrosis more effectively.

## 3. Discussion

We investigated the pathological role of IS on renal fibrosis and its underlying mechanisms using *Sult1a1*-KO mice. *Sult1a1*-KO mice exhibited reduced renal fibrosis, inflammation, and apoptosis and improved EPO production. CD206^+^ macrophage infiltration prevents the progression of inflammation and renal fibrosis. The inactivation of Wnt/β-catenin signaling is involved in decreasing renal fibrosis; treating *Sult1a1*-KO mice with rhEPO further attenuated UUO-induced renal fibrosis, indicating the possibility of combination therapy in patients with CKD.

IS accumulation has been linked to CKD progression. In an ultra-performance LC-MS/MS (UPLC-MS/MS) analysis, serum IS in healthy participants was ≤0.05–3.02 mg/L. The average IS level progressively increased from 1.03 mg/L in CKD stage 1 to 12.21 mg/L in CKD stage 5 [21]. However, there was no significant increase in serum BUN in the WT-UUO group (Figure 1A); *Sult1a1* mRNA expression and serum IS concentration were elevated in the WT UUO group (Figure 1B,C). IS administration (100 mg/kg/day, intraperitoneal) to healthy mice for 3 days has been reported to increase significantly serum and kidney IS concentrations within 3 h of the last administration [22], which indicates that IS is briefly retained in the serum and kidneys, even in normal renal function.

In addition, human sulfotransferase SULT1A1 is a crucial phase II xenobiotic-metabolizing enzyme that plays a vital role in sulfonating drugs, carcinogens, and steroids; it is highly expressed in the liver. The human sulfotransferase SULT1A1 is regulated by specificity protein 1 (Sp1) and GA-binding protein (GABP). Elevated IS levels affect Sp1- or GA-binding proteins and increase *Sult1a1* transcript levels in the liver [23]. Therefore, the decreased elimination from UUO-treated kidneys may have caused a vicious cycle of increased *Sult1a1* transcription levels in the liver due to the accumulation of serum IS. Moreover, IS administration in animal CKD models increased IS retention in renal tubular cells and was accompanied by cell death in OAT1- and OAT3-expressing proximal tubular cells [24]. This effect could be rescued by probenecid, an anion transport inhibitor. Thus, inadequate renal clearance of IS during renal function decline may further aggravate IS-induced renal tubule cytotoxicity and accelerate CKD progression. Our results confirmed IS accumulation in the UUO mouse model; we hypothesized that the accumulated IS further affected renal fibrosis. Previous research established that combining benazepril (used to treat diabetic kidney disease) and AST-120 (which absorbs the precursor of IS in the intestine) reduces the progression of renal fibrosis in uremic rats [25], which is consistent with our results in that a decrease in IS accumulation reduces renal fibrosis. In addition, IS activates mTORC1- and adenine-induced renal fibrosis, which was attenuated by AST-120 treatment [22]. All reports indicated that IS is related to renal fibrosis by several signal activation, consistent with our results; however, different signaling pathways are involved due to the various stimuli to the kidneys.

Animal models suggest that the primary causes of renal fibrosis are uncontrolled epithelial damage and inflammation [26]. To investigate this, we examined the expression of inflammatory cytokines in WT and *Sult1a1*-KO mice kidneys. IL-6, TNF-α, and IL-1β levels increased in WT-UUO mice but decreased in *Sult1a1*-KO UUO mice, indicating that IS accumulation promotes inflammation in UUO kidneys. *Sult1a1*-KO UUO mice exhibited infiltrated CD206^+^ macrophages, which could play an anti-inflammatory role. During inflammation, infiltrating leukocytes activate intrinsic renal cells, releasing profibrotic cytokines and growth factors, which leads to myofibroblast recruitment and activation, resulting in progressive renal fibrosis [27]. Therefore, we hypothesized that CD206^+^ macrophages may inhibit ongoing inflammation and subsequent renal fibrosis via profibrotic cytokines and growth factor recruitment decrease.

On the other hand, a genome-wide transcriptome investigation of kidneys from patients with CKD and fibrosis revealed differential expression of genes associated with the Notch, Wnt, and Hedgehog signaling pathways compared to healthy individuals [28]. Additionally, after ischemia-reperfusion (IR) injury in mice, elevated Wnt ligand expression in isolated macrophages and an enhanced Wnt signaling response in epithelial cells were observed [29]. Wnt4 promotes tubular epithelial cell regeneration by regulating the cell-cycle proteins cyclin D1 and cyclin A [30]. Furthermore, lineage-tracing studies in mice revealed that Wnt4 expression was high in activated myofibroblasts, particularly in the medullary interstitium after UUO or IR injury [28]. In our study, the expression of Wnt4 was upregulated in the UUO model, and *Sult1a1*-KO demonstrated no effect on Wnt4 expression, suggesting that Wnt4 is independent of IS-related renal fibrosis.

Additionally, *Sult1a1*-KO mice exhibited elevated levels of Sfrp5, which inhibits the Wnt signaling pathway by binding to the Wnt protein. According to Yanlin Yu et al., IS increased renal fibrosis by downregulating sfrp5 expression and activating the Wnt/β-catenin signaling pathway [31]. This result is consistent with our study, confirming that sfrp5 is involved in IS-induced renal fibrosis. Therefore, the up-regulation of sfrp5 in *Sult1a1*-KO mice suppressed β-catenin activation, resulting in the prevention of fibrosis marker expression.

EPO is known to regulate the formation of red blood cells by stimulating bone marrow and is widely used for the clinical treatment of anemia [32,33]. However, EPO unresponsiveness in several patients is common, suggesting that undefined causes affect anemia [34]. Uremic toxin accumulation in blood impairs erythropoietin synthesis by compromising the growth and differentiation of red blood cells in the bone marrow, leading to impaired erythropoiesis [35]. IS impairs erythropoiesis by triggering apoptosis and senescence [34]. Our results revealed elevated EPO production in *Sult1a1*-KO mice (Figure 6A). Therapy targeting *Sult1a1* may demonstrate the ability to treat UUO-related anemia. Recent studies discovered that EPO might efficiently protect against renal failure [36]. EPO significantly enhanced the recovery from acute renal failure induced by cisplatin in rats [37]. In rats with IR of the kidney, rhEPO protected renal function and structure and reduced fibrosis and myofibroblast stimulation [38]. Consistently, our results indicate that *Sult1a1*-KO partly improved EPO production and progressive renal fibrosis was further suppressed by the administration of rhEPO to *Sult1a1*-KO mice. Although the treatment of EPO against renal fibrosis is under investigation, a high dose of EPO was found to contribute to fibrogenesis in the long term [38]. Combining EPO treatment with IS suppression therapy may be a potential solution.

The mechanism through which IS activates inflammatory responses in the UUO model is unknown. However, IS is known to be a potent inducer of free radicals and may cause oxidative stress in the renal and cardiovascular systems [13,39]. Accordingly, we hypothesized that ureter obstruction causes IS accumulation, which can harm tubular cells by inducing oxidative stress, leading to the release of inflammatory cytokines. In addition, protein-bound uremic toxins contain 25 compounds [40]. Other than IS, the accumulation of PCS, produced mainly by SULT1A1 in the liver, decreased kidney function in patients with CKD [41]. Its effect on renal fibrosis would be investigated using *Sult1a1*-KO mice in the future.

**Figure 7:**
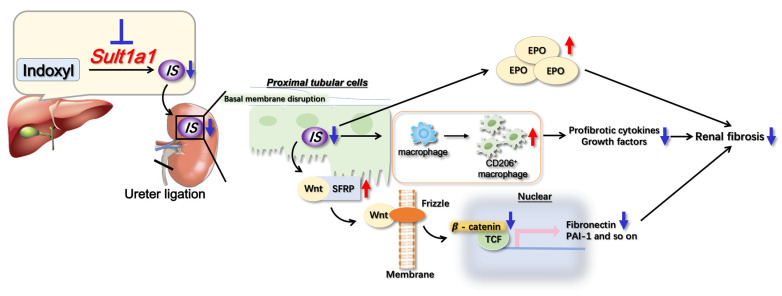
Summary of the mechanisms underlying UUO-induced renal fibrosis suppressed in *Sult1a1*-KO mice. After the unilateral ureter was obstructed in *Sult1a1*-KO mice, IS accumulation was diminished in the serum and renal tissues due to a lack of *Sult1a1* activity. UUO-induced renal fibrosis was suppressed, accompanied by decreased IS concentration. Furthermore, *Sult1a1* inhibition induced the infiltration of CD206^+^ macrophages, suppressing ongoing inflammation and, as a result, renal fibrosis. Inactivated Wnt/β-catenin signaling and increased EPO production also help to reduce renal fibrosis.

In conclusion, *Sult1a1*-KO mice demonstrated less IS build-up, which reduced UUO-induced renal fibrosis; infiltrating CD206^+^ macrophages may aid inflammation and suppress renal fibrosis in *Sult1a1*-KO mice. Additionally, the inactivation of Wnt/β-catenin signaling helped slow the progression of renal fibrosis. Furthermore, in *Sult1a1*-KO mice, rhEPO administration suppressed renal fibrosis more effectively. Our findings confirmed the direct pathological role of IS in kidney inflammation and fibrosis, identifying SULT1A1 as a new therapeutic target for preventing or attenuating renal fibrosis (summarized in Figure 7).

## 4. Materials and Methods

### 4.1. Sult1a1-KO Mice

*Sult1a1*-deficient mouse embryos (Deltagen, San Carlos, CA, USA) were purchased, melted, and transplanted into an expedient parent at the Kumamoto University Institute of Resource Development and Analysis (IRDA) to manufacture the heteromouse. The mice were bred in the Animal Resources and Development (CARD) of Kumamoto University. Homogenized mice were then manufactured.

### 4.2. Animal Experiments

All procedures for animal experiments were approved by the Kumamoto University ethical committee concerning animal experiments (Identification code: A 2023-034, Approval date: 1 April 2023) and were treated in accordance with the Guidelines of the United States National Institutes of Health regarding the care and use of animals for experimental procedures and the Guidelines of Kumamoto University for the care and use of laboratory animals.

In this study, we have conducted three animal experiments. In protocol 1, we established a 2-week-UUO model on WT and *Sult1a1*-KO mice to investigate the pathological role of IS in UUO-induced renal fibrosis. C57BL/6J male mice (WT, 8-week-old) and *Sult1a1*-KO mice (8-week-old) were randomized and anesthetized before unilateral ureteral obstruction (UUO) treatment. WT and *Sult1a1*-KO mice were classified into four groups containing 3–9 mice each: WT Sham, WT UUO, *Sult1a1*-KO, and *Sult1a1*-KO UUO groups. The left ureter of each mouse was ligated with 3-0 silk, and the abdomen was closed with sutures. After surgery, the mice were warmed until recovery. The sham animals (control) underwent anesthesia and laparotomy only. The mice were sacrificed on day 14 after surgery.

In protocol 2, we evaluated the effect of *Sult1a1* knockout on changes in erythropoietin (EPO) production capacity over time. Group classification and UUO surgery were conducted as described in protocol 1. group classification and UUO surgery were conducted as described in protocol 1. Each group contained 3–8 mice. To confirm the time-related changes in EPO levels in WT and *Sult1a1*-KO mice, mice were sacrificed on days 0, 1, 3, 7, and 14 following UUO surgery.

In protocol 3, recombinant human erythropoietin (rhEPO) was administered to WT and *Sult1a1*-KO mice to investigate further the relationship between IS, EPO, and renal fibrosis. C57BL/6J male mice (WT, 8-week-olds) and *Sult1a1*-KO mice (8-week-olds) were randomized and anesthetized before UUO treatment. WT and *Sult1a1*-KO mice were classified into six groups: WT Sham, WT UUO, and WT UUO+EPO; *Sult1a1*-KO Sham, *Sult1a1*-KO UUO, and *Sult1a1*-KO UUO+EPO. Each group contained five mice. UUO surgery was performed as described in Protocol 1. RhEPO (R&D Systems, Minneapolis, MN, USA) was diluted to 12.5 U/mL in sterile PBS (containing 0.1% bovine serum albumin) and administered intraperitoneally at 8 mL/kg every other day starting on day 3 after UUO treatment. Sterile PBS containing 0.1% bovine serum albumin was used as the control. The mice were sacrificed on day 14 after surgery.

### 4.3. Liquid Chromatography/Mass Spectrometry/MS (LC-MS/MS) Assay of IS Concentration

Samples were processed with acetonitrile, vortexed, and centrifuged to remove the protein. IS concentrations were measured using an API 3200TM LC-MS/MS system (AB SCIEX, Foster City, CA, USA) with a triple quadruple mass spectrometer following negative ion mode: IS, m/z 212.08. The detailed settings of LC/MS/MS were performed as previously described [16].

### 4.4. Sirius Red Staining

Sirius red staining was performed for collagen deposition analysis. The tissue of mouse kidneys harvested on day 14 after obstruction was fixed in 10% phosphate-buffered formaldehyde for 48 h, embedded in paraffin, sectioned (4 μm), and deparaffinized. Adequate picrosirius red solution was applied to cover the tissue section completely and incubated for 60 min. The slides quickly rinsed with two changes of 0.5% acetic acid solution. The slides were then rinsed using absolute alcohol, dehydrated in two changes of absolute alcohol, cleared, and mounted in synthetic resin. The percentage of collagen deposition area versus the whole tissue area was calculated using an all-in-one fluorescence microscope BZ-X700 (×200 magnification) (KEYENCE, Osaka, Japan). Five areas in each section were calculated for each mouse. Data from the mice in the same treatment groups were averaged.

### 4.5. TUNEL Staining

Kidney specimens from WT and *Sult1a1*-KO mice were stained using an in situ apoptosis detection kit (Takara Bio Inc., Kusatsu, Japan). Under light microscopy (×200 magnification), the number of TUNEL-positive cells in the area covering the majority of corticomedullary junctions in a slide was counted by researchers blinded to the samples. The number of TUNEL-positive cells in five sections from each mouse was counted. Data from the mice in the same treatment groups were averaged.

### 4.6. CD206 Immunostaining

Formalin-fixed paraffin-embedded kidneys were deparaffinized and the endogenous peroxidase activity was quenched. The primary antibody CD206 (MR5D3, Thermo Fisher Scientific, Waltham, MA, USA) was applied, followed by staining with Goat Anti-Rat IgG H&L (ab97057, abcam, Tokyo, Japan). The CD206^+^ area was calculated using an all-in-one fluorescence microscope BZ-X700 (×200 magnification) (KEYENCE). Five areas in each section were calculated for each mouse. Data from the mice in the same treatment groups were averaged.

### 4.7. Western Blot Analysis

Western blot analysis was performed as previously described [16]. The total protein from kidney issues was extracted with RIPA buffer and Western blot analysis was completed with antibodies as follows: α-SMA (ab5694, abcam), β-catenin (ab32572, abcam) or GAPDH (ab199713, abcam). The dilutions of the antibodies are presented in Table 1. The dilution agent used was 5% *v*/*v* nonfat dry milk.

### 4.8. Real-Time PCR

Total RNA was isolated from the kidneys using TRIzol (Invitrogen, Tokyo, Japan) according to the manufacturer’s protocol. The cDNA synthesis and PCR amplifications were performed as previously described [16]. The primers (NIHON GENE RESEARCH LABORATORIES Inc., Sendai, Japan) used for the mice are presented in Table 2. The threshold cycle (Ct) values for each gene amplification were normalized by subtracting the Ct value for GAPDH. The normalized gene expression values are expressed as the relative quantity of gene-specific mRNA compared to GAPDH.

### 4.9. Statistical Analysis

Data were statistically analyzed by analysis of variance, followed by Tukey’s or Scheffé’s multiple comparisons test, using Statcel2 software (OMS Ltd., Saitama, Japan). Statistical *p*-value < 0.05 was considered significant. All data are presented as mean ± standard deviation (SD).

## Figures and Tables

**Table 1:** Dilution of antibody.

Target Protein	The Primary Antibody	The Secondary Antibody
α-SMA	Dilution: 5000×	Dilution: 20,000×
β-catenin	Dilution: 5000×	Dilution: 10,000×
GAPDH	Dilution: 5000×	Dilution: 5000×

**Table 2:** Primer sequences for each gene.

Gene	Forward (5′-3′)	Reverse (5′-3′)
Sult1a1	TGAGACGCACTCACCCTGTTCT	TCCACAGTCTCCTCAGGTAGAG
Col1a1	GACAAATGAATGGGGCAAG	CAATGTCCAGAGGTGCAATG
IL-6	TACCACTTCACAAGTCGGAGGC	CTGCAAGTGCATCATCGTTGTTC
TNF-α	CTACCTTGTTGCCTCCTCTTT	GAGCAGAGGTTCAGTGATGTAG
IL-1β	AGTTGACGGACCCCAAAAG	AGCTGGATGCTCTCATCAGG
F4/80	CGTGTTGTTGGTGGCACTGTGA	CCACATCAGTGTTCCAGGAGAC
CD206	GTTCACCTGGAGTGATGGTTCTC	AGGACATGCCAGGGTCACCTTT
Wnt4	GCGTAGCCTTCTCACAGTCC	CGCATGTGTGTCAAGATGG
sFRP5	GATCTGTGCCCAGTGTGAGA	TATGCAGGACCAGCTTCTTGGTGT
β-catenin	GCAGCAGCAGTCTTACTTGG	CCCTCATCTAGCGTCTCAGG
Fibronectin	AGACCATACCTGCCGAATGTAG	GAGAGCTTCCTGTCCTGTAGAG
EPO	CACAACCCATCGTGACATTTTC	CATCTGCGACAGTCGAGTTCTG
GAPDH	CGACTTCAACAGCAACTCCCACTCTTCC	TGGGTGGTCCAGGGTTTCTTACTCCTT

## Data Availability

Data available upon request.
